# University of Pennsylvania

**Published:** 1860-07

**Authors:** 


THE UNIVERSITY OF PENNSYLVANIA.—The Chair of Medicine in this Institution, rendered vacant by the retirement of Professor Wood, was filled early in the past month by the appointment of Dr. William Pepper, of this city, well known to the profession as a distinguished practitioner and clinical lecturer.

